# Early Initiation of Anticoagulation Improves the Long-Term Prognosis in Patients With Antiphospholipid Syndrome Associated Portal Vein Thrombosis

**DOI:** 10.3389/fmed.2021.630660

**Published:** 2021-02-04

**Authors:** Hanxiao You, Jiuliang Zhao, Can Huang, Xinping Tian, Mengtao Li, Xiaofeng Zeng

**Affiliations:** Department of Rheumatology, Peking Union Medical College Hospital, Peking Union Medical College & Chinese Academy of Medical Science, National Clinical Research Center for Dermatologic and Immunologic Diseases, Ministry of Science & Technology, Key Laboratory of Rheumatology and Clinical Immunology, Ministry of Education, Beijing, China

**Keywords:** antiphospholipid syndrome, portal vein thrombosis, anticoagulation, portal hypertension, thrombosis

## Abstract

**Objectives:** Portal vein thrombosis (PVT) is a rare and severe clinical phenotype of antiphospholipid syndrome (APS) with a poor prognosis. Anticoagulation therapy is efficient but is associated with potentially severe bleeding episodes, especially for those patients with thrombocytopenia. We conducted this case-control study to explore the clinical features and associated factors of PVT in APS patients, the re-canalization rate of the PVT after anticoagulation and investigate the beneficial effects of early initiation of anticoagulation in patients with APS associated PVT.

**Methods:** We enrolled patients with APS associated PVT as the case group, and age-, and entry-time-matched APS patients without PVT (1:2) as the control group. We explored the associated factors of PVT in APS patients using multivariate logistic regression analysis. The re-canalization rate of the PVT after anticoagulation was analyzed using the survival analysis.

**Results:** A total of 34 patients (8 males and 26 females) with APS-PVT were enrolled, with a median follow-up time of 3 years (1.5, 7 years). Multivariate logistic regression analysis showed that thrombocytopenia (OR 6.4, 95%CI 1.561–26.218, *P* = 0.01), hypersensitive c-reactive protein >3 mg/L (OR 4.57, 95%CI 1.426–14.666, *P* = 0.011), anti β2GPI positive (OR 5, 95%CI 1.816–13.772, *P* = 0.002) and aPL double-positive (OR 4.08, 95%CI 1.312–12.429, *P* = 0.013) were independent associated factors for PVT in APS. Survival analysis revealed that effective anticoagulation could increase re-canalization rate significantly (log-rank *p* = 0.001), with better prognosis (lower mortality rate, log-rank *p* = 0.045).

**Conclusions:** PVT could be the first presentation of APS with insidious onset and atypical clinical symptoms and easily be misdiagnosed. For patients with APS, double aPLs positive, thrombocytopenia, and inflammation could be the associated factors of PVT. Early diagnosis and anticoagulation treatment can bring thrombus re-canalization thereby significantly improving the prognosis.

## Key-Points

- PVT could be the first thrombotic event of APS, usually had insidious onset with atypical clinical symptoms and easily be misdiagnosed.
- For patients with APS, the double aPLs positive, thrombocytopenia, and inflammation could be the risk factor of PVT.
- Early diagnosis and anticoagulation treatment can bring thrombus re-canalization thereby significantly improving the prognosis, with a lower mortality rate.

## Introduction

Antiphospholipid syndrome (APS) is a systemic autoimmune disease characterized by recurrent arterial venous thrombosis, habitual abortion, and/or thrombocytopenia and persistent antiphospholipid antibodies (aPLs) positive in the blood. PVT is a rare, serious, and highly heterogeneous phenotype of APS (1). Portal venous system thrombosis (PVT) includes the thrombus in the portal vein, the superior mesenteric vein/splenic vein, and the inferior mesenteric vein. According to the course of thrombosis, portal vein thrombosis is divided into acute PVT, chronic PVT, and portal vein degeneration. Some patients had insidious onset with atypical clinical symptoms and easily be misdiagnosed. PVT has been related to liver dysfunction, neoplasm, genetic factors (2, 3), hemodynamic factors, and hypercoagulability states (4). Antiphospholipid antibodies also have been implicated as one of the causes of PVT in a few previous studies (5, 6).

It is recommended that anticoagulation should be given for 3–6 months if acute PVT was detected early. If prothrombotic factors are identified, anticoagulation should be given lifelong (7). Anticoagulation therapy is efficient, however, may be associated with potentially severe harmful effects, especially bleeding episodes for those patients with thrombocytopenia. Also, the literature lacks information about the safety and long-term results of anticoagulation in patients of APS associated PVT. Most clinical evidence and treatment experience about PVT came from PVT caused by abdominal infections, tumors, or cirrhosis. Only a few studies have been reported about PVT due to APS. And most are only case reports (8–10), there is only one cross-sectional study of 32 patients (11). This study aimed to explore the clinical features and associated factors of PVT in APS patients, the re-canalization rate of the PVT after anticoagulation, and the beneficial effects of early initiation of anticoagulation in patients with APS associated PVT.

## Methods

### Patient Recruitment

We utilized the Hospital Inpatient Information Retrieval System to identify the patients with APS associated PVT admitted to the Peking Union Medical College Hospital (PUMCH) from January 2012 to December 2019. A case-control study was conducted to explore the clinical features and associated factors of PVT in APS patients. Patients with APS associated PVT were defined as the case group, and age-, sex-, and entry-time-matched APS patients without PVT at the ratio of 1:2 as the control group. The diagnosis of APS was confirmed according to the 2006 Sydney classification criteria for definite antiphospholipid syndrome (12) and PVT was confirmed according to the 2009 American College of Liver Diseases (AASLD) criteria. An acute PVT was defined if symptoms developed <60 days before hospital assessment and the absence of or insignificant portal collaterals on imaging and no evidence of portal hypertension including splenomegaly and oesophageal varices (7). Patients with alcoholic cirrhosis, viral hepatitis induced postnecrotic cirrhosis, tumorous obstruction, and patients with a history of abdominal surgery before PVT were excluded. Medical records were reviewed for medical history, results of antiphospholipid antibodies (aPL) testing, including lupus anticoagulant (LAC), anticardiolipin (ACL), and anti-β2-glycoprotein I (antiβ2GPI), IgG or IgM autoantibodies. Subjects were considered aPL positive if at least one of these autoantibodies, at least 12 weeks apart were documented. IgG/IgM antibody of ACL and anti-β2-glycoprotein I were tested by enzyme linked immunosorbent assay (ELISA). We considered positive those titers reported as medium (20–40) or high (>40), being low titers considered as negative. Dilute Russell viper venom time (dRVVT) testing and activated partial thromboplastin time were measured, where LAC was considered positive if the ratio of dRVVT time >1.20. Regular imaging was performed to monitor the outcome of PVT. The data of the re-canalization rate of the PVT after anticoagulation were also collected. The date of entry was the date of the first diagnosis of PVT. Patients were followed every 3–6 months until death, or the end of the study (July 2019). Effective anticoagulation was defined as immediate and sufficient anticoagulant therapy for at least 6 months with a target international normalized ratio (INR) of 2.0–3.0 from the time of PVT diagnosis. This study was approved by the Medical Ethics Committee of the Peking Union Medical College Hospital, which was the lead site, and all patients provided written informed consent.

### Statistical Analysis

Continuous variables were presented as means and standard deviation (SD) for normally distributed data, and medians and interquartile range (IQR) (P25, P75) for all other data, whereas categorical variables were presented as number (percentage). In bivariate analysis, continuous variables were compared with the use of the Student's *t*-test or Mann–Whitney test, while categorical variables were compared using the chi-square test or Fisher's exact test. Variables were entered into the univariable (UV) logistic regression model. A multivariate (MV) logistic regression model was then constructed using a stepwise forward selection procedure among those candidate variables with the significance level *p* < 0.10 in the UV logistic regression analysis. Continuous variables are converted to binary or ordered multiple variables when entered into UV or MV logistic regression models. Odds ratios and 95% confidence intervals were calculated. Survival analysis using the log-rank test was applied to compare the accumulated recanalization rate between effective and invalid anticoagulation groups. The *p*-value was two-tailed and defined as significant if the value was <0.05. SPSS software, version 23 (Chicago, IL, USA) used for all the statistical descriptions, analyses, and inferences.

## Results

### Clinical Manifestations

A total of 34 patients confirmed with PVT from 187 APS patients were identified, of which 18 patients were primary APS, 14 patients were secondary to systemic lupus erythematosus (SLE), and 2 patients were secondary to Sjogren's syndrome. Sixty-eight age-, sex-, and entry-time-matched APS patients without PVT were selected as the control group, of which 32 patients were primary APS, and 36 patients were secondary to SLE. Comparison of demographic characteristics, clinical, and laboratory manifestations between the case (APS patients with PVT) and control (APS patients without PVT) groups were shown in Table 1. There were no significant differences in gender, age at study entry between the case and control groups. In the case group, there were 8 males and 26 females, with a mean age of 40.35±13.029 years, disease course 0.08 years (0, 1.13 years), and median follow-up was 3 years (1.5, 7years). Triple aPLs were positive in 7 cases and double aPLs positive in 15 cases. 11 cases were acute thrombosis, 23 cases chronic thrombosis, and 7 cases portal vein cavernoma. Among the case group, thrombosis of portal veins was initially demonstrated using Doppler ultrasound in 14 cases and computed tomography angiography in 20 cases.

**Table 1:** Comparison of demographic characteristics, clinical and laboratory manifestations between APS patients with and without PVT.

**Variable**	**APS patients with PVT (*n* = 34)**	**APS patients without PVT (*n* = 68)**	***p*-value**
Female, *n* (%)	26 (76.5)	54 (79.4)	0.734
Age, mean (SD), years	40.35 ± 13.029	40.25 ± 13.453	0.97
Disease course, median (P25, P75), years	0.08 (0, 1.13)	4 (1, 9.75)	** <0.001**
Follow-up time, median (P25, P75), years	3 (1.5, 7)	1 (0, 4)	0.861
BMI, median (P25, P75), kg/m^2^	21.36 (18.55, 25.28)	22.43 (20.73, 25.95)	0.788
Arterial thrombosis, n (%)	6 (17.6)	26 (38.2)	0.035
Venous thrombosis, n (%)	34 (100)	19 (27.9)	** <0.001**
Pregnancy loss, n (%)	1 (2.9)	15 (22.1)	0.018
PAPS, n (%)	18 (52.9)	32 (47.1)	0.575
SLEDAI (for SLE-related APS), median (P25, P75)	5 (1, 10) (*n* = 14)	2 (0, 2) (*n* = 36)	**0.004**
SLICC (for SLE-related APS), median (P25, P75)	1 (0, 2) (*n* = 14)	0 (0, 1) (*n* = 36)	0.196
Thrombocytopenia, *n* (%)	11 (32.4)	6 (8.8)	**0.003**
Hypoalbuminemia, *n* (%)	16 (47.1)	1 (1.5)	** <0.001**
ESR >20 mm/h, *n* (%)	12 (35.3)	14 (20.6)	0.108
hsCRP>3 mg/L, *n* (%)	15 (44.1)	9 (13.2)	**0.001**
Total cholesterol >5.7 mmol/L, *n* (%)	5 (14.7)	2 (2.9)	**0.027**
Total triglycerides>1.7 mmol/L, *n* (%)	6 (17.6)	10 (14.7)	0.7
ACL positive, *n* (%)	20 (58.8)	41 (60.3)	0.886
anti β2GPI positive, *n* (%)	24 (70.6)	20 (29.4)	** <0.001**
LAC positive, *n* (%)	19 (55.9)	36 (52.9)	0.779
aPL double positive, *n* (%)	15 (44.1)	17 (25)	**0.05**
aPL triple positive, n (%)	7 (20.6)	17 (25)	0.62

*Values in bold are statistically significant at p < 0.05*.

*APS, antiphospholipid syndrome; PAPS, primary antiphospholipid syndrome; PVT, portal vein thrombosis; SD, standard deviation; BMI, body mass index; SLE, systemic lupus erythematosus; SLEDAI, SLE disease activity index; ESR, erythrocyte sedimentation rate; hsCRP, hypersensitive C-reactive protein; LAC, lupus anticoagulant; ACL, anticardiolipin; antiβ2GPI, anti-β2-glycoprotein I*.

PVT could be the first presentation of APS with insidious onset and atypical clinical symptoms. PVT was the first thrombotic event of APS in 21 patients; 2 patients had other types of thrombosis (myocardial infarction, deep venous thrombosis, respectively) as the first event; and 11 patients presented as thrombocytopenia first. As for the first symptoms of PVT, the presentation with abdominal distention (14 cases) and pain (10 cases) were more common, the presentation with variceal bleeding (5 cases) was less common, and 7 cases were asymptomatic. Among the case group, 17 cases were complicated with portal hypertension. Eighteen cases had dilated esophageal or gastric veins, 22 cases with splenomegaly, 5 cases of splenectomy, and 9 cases of liver cirrhosis (Table 2). Four of these 34 patients presented with intestinal infarction, 3 of whom required an intestinal resection.

**Table 2:** The clinical features, treatment and prognosis of APS patients with PVT.

	***n* (*N* = 34)**	**%**
**Stage at recognition**		
Acute	11	32.35
Chronic	23	67.65
**The first symptom of APS**		
PVT	21	61.76
Other types of thrombosis	2	5.88
Thrombocytopenia	11	32.35
**The first symptom of PVT**		
Abdominal pain	10	29.41
Abdominal distention	14	41.18
Gastrointestinal bleeding	5	14.71
Asymptomatic	7	20.59
**Complications**		
Portal hypertension	17	50.00
Dilated esophageal or gastric veins	18	52.94
Splenomegaly	22	64.71
Splenectomy	5	14.71
Liver cirrhosis	9	26.47
**Treatment**		
Effective anticoagulation	16	47.06
Invalid anticoagulation	13	38.24
No anticoagulation	5	14.71
**Outcomes**		
Complete re-canalization	7	20.59
Partial re-canalization	10	29.41
No re-canalization	12	35.29
Death	5	14.71

*APS, antiphospholipid syndrome; PVT, portal vein thrombosis*.

Multivariate logistic regression analysis showed that thrombocytopenia (OR 6.4, 95%CI 1.561–26.218, *P* = 0.01), hypersensitive c-reactive protein >3 mg/L (OR 4.57, 95%CI 1.426–14.666, *P* = 0.011), anti β2GPI positive (OR 5, 95%CI 1.816–13.772, *P* = 0.002), and aPLs double positive (OR 4.08, 95%CI 1.312–12.429, *P* = 0.013) were independently associated factors for PVT in APS, as shown in Table 3.

**Table 3:** Univariate and multivariate logistic regression analyses for variables predictive of PVT in APS patients.

**Variables**	**UV**		**MV**	
	**OR (95% CI)**	***P*-value**	**OR (95% CI)**	***P*-value**
BMI>25 kg/m^2^	0.64 (0.25, 1.65)	0.358		
PAPS	1.27 (0.56, 2.89)	0.576		
Thrombocytopenia	4.94 (1.64, 14.9)	**0.005**	6.40 (1.56, 26.22)	**0.01**
ESR >20 mm/h	2.1 (0.84, 5.26)	0.112		
hsCRP>3 mg/L	5.18 (1.95, 13.72)	**0.001**	4.57 (1.43, 14.67)	**0.011**
Total cholesterol >5.7 mmol/L	5.69 (1.04, 31.05)	**0.045**		
Total triglycerides>1.7 mmol/L	1.24 (0.41, 3.76)	0.701		
ACL positive	0.94 (0.41, 2.18)	0.886		
Anti β2GPI positive	5.76 (2.33, 14.22)	** <0.001**	5.00 (1.82, 13.77)	**0.002**
LAC positive	1.13 (0.49, 2.58)	0.779		
aPL double positive	2.37 (0.99, 5.66)	0.052	4.08 (1.34, 12.43)	**0.013**
aPL triple positive	0.78 (0.29, 2.11)	0.621		

*Values in bold are statistically significant at p < 0.05*.

*UV, Univariate; MV, multivariate; OR, odds ratio; CI, confidence interval; BMI, body mass index; PAPS, primary antiphospholipid syndrome; ESR, erythrocyte sedimentation rate; hsCRP, hypersensitive C-reactive protein; LAC, lupus anticoagulant; ACL, anticardiolipin; antiβ2GPI, anti-β2-glycoprotein I*.

### Treatment and Prognosis

Twenty-nine of 34 patients received anticoagulation therapy, five patients did not receive anticoagulation treatment because of a high risk of gastrointestinal bleeding (Table 3). Sixteen cases began effective anticoagulation therapy immediately at the diagnosis of thrombus and for at least 6 months. Anticoagulation therapy consisted of the initial administration of intravenous unfractionated heparin in 29 patients, with a median duration of 19 days (range, 7–60 days), followed by oral anticoagulation in all patients, 25 patients with warfarin and 4 patients with rivaroxaban. Rhinorrhea occurred in one of the patients receiving anticoagulation. No surgical thrombectomy was performed. No thrombolytic therapy was administered. Among the 34 patients, 7 patients got thrombus complete recanalization and 10 patients partial recanalization, 5 patients died.

**Figure 1:**
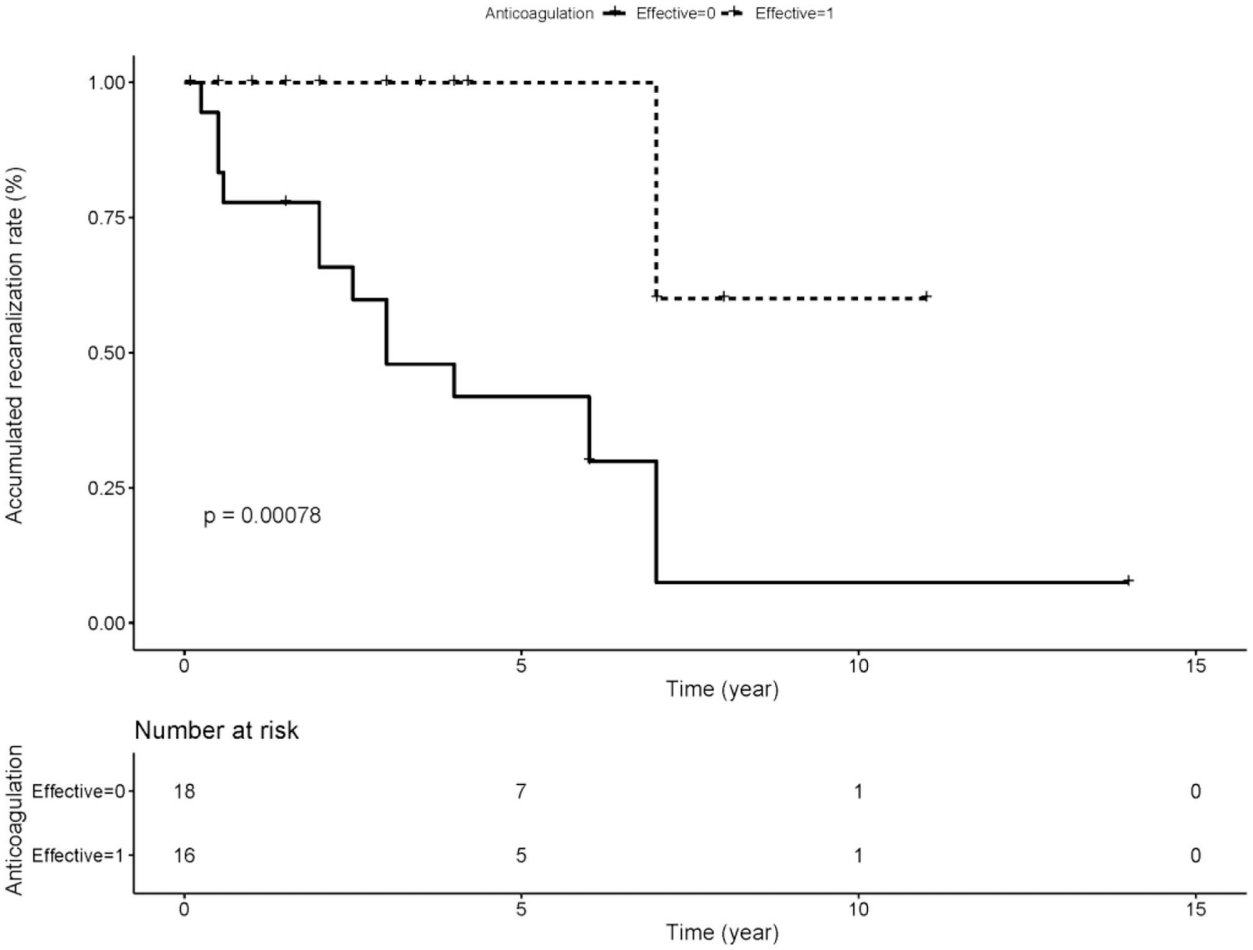
Difference of accumulated re-canalization rate between groups receiving effective anticoagulation (*n* = 16) and not (*n* = 18). Effective anticoagulation was defined as immediate and sufficient anticoagulant therapy for at least 6 months with a target international normalized ratio (INR) of 2.0–3.0 from the time of portal vein thrombosis (PVT) diagnosis.

**Figure 2:**
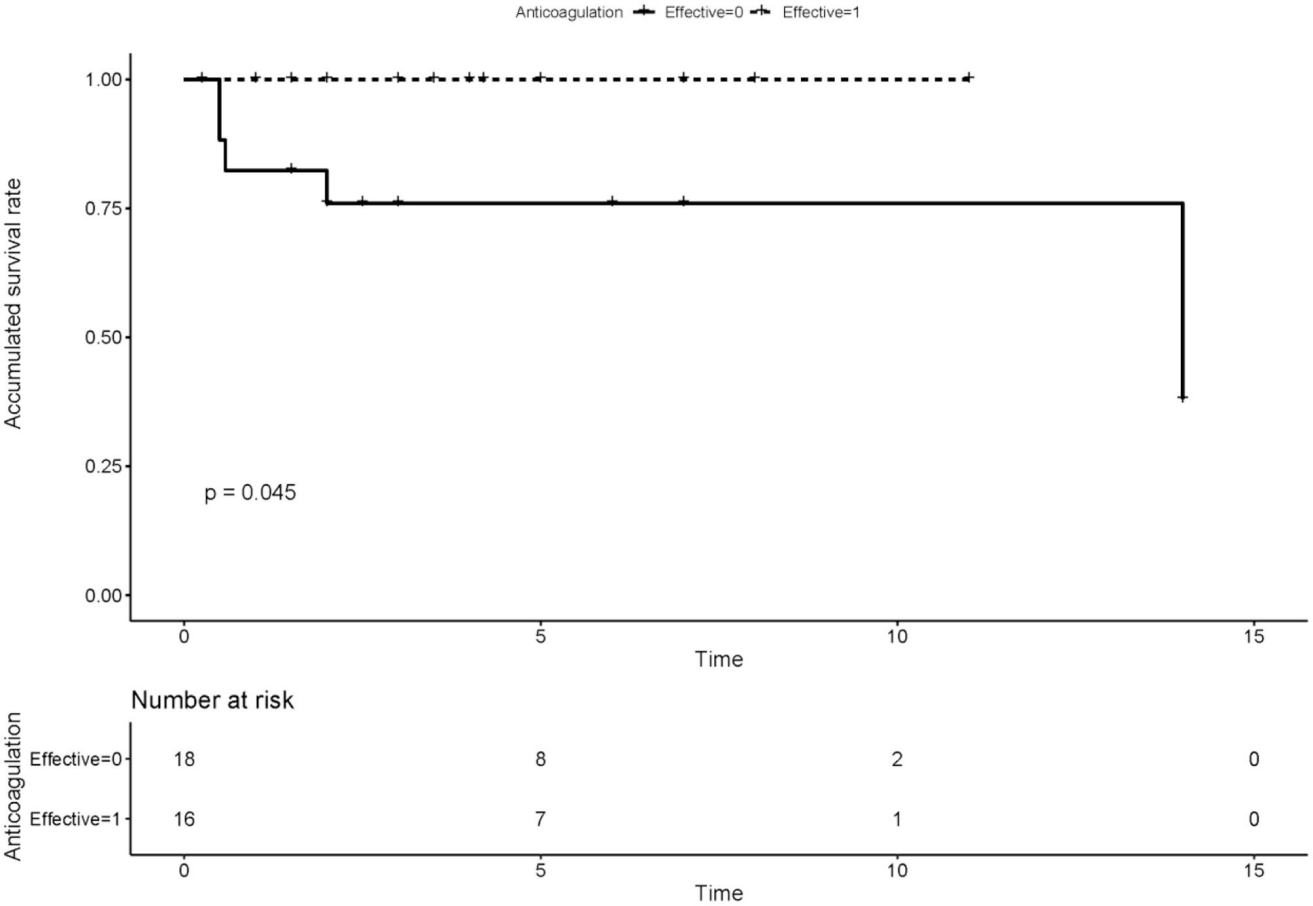
Difference of accumulated survival rate between groups receiving effective anticoagulation (*n* = 16) and not (*n* = 18). Effective anticoagulation was defined as immediate and sufficient anticoagulant therapy for at least 6 months with a target international normalized ratio (INR) of 2.0–3.0 from the time of portal vein thrombosis (PVT) diagnosis.

**Figure 3:**
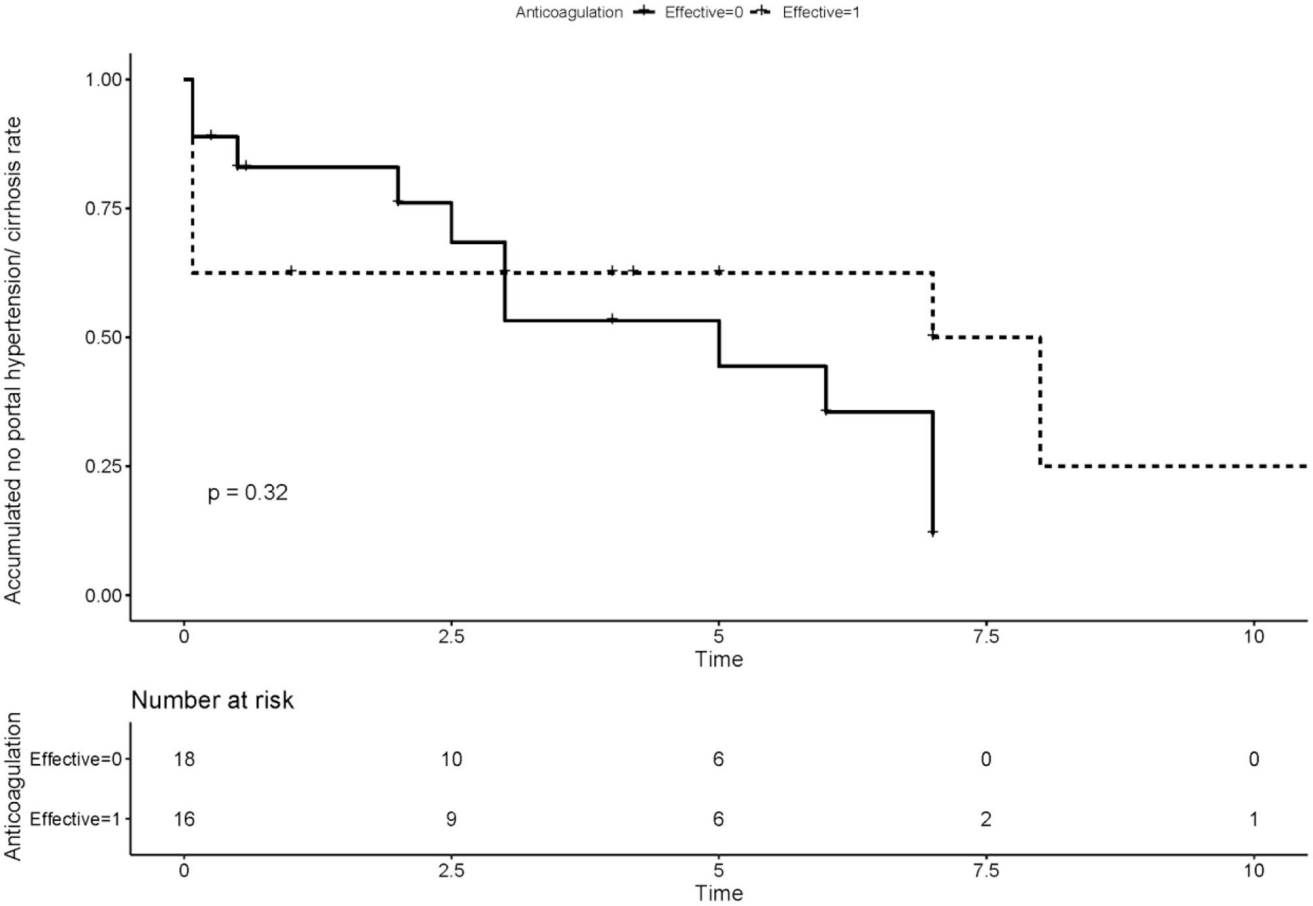
Difference of accumulated no portal hypertension/cirrhosis rate between groups receiving effective anticoagulation (*n* = 16) and not (*n* = 18). Effective anticoagulation was defined as immediate and sufficient anticoagulant therapy for at least 6 months with a target international normalized ratio (INR) of 2.0–3.0 from the time of portal vein thrombosis (PVT) diagnosis.

**Figure 4:**
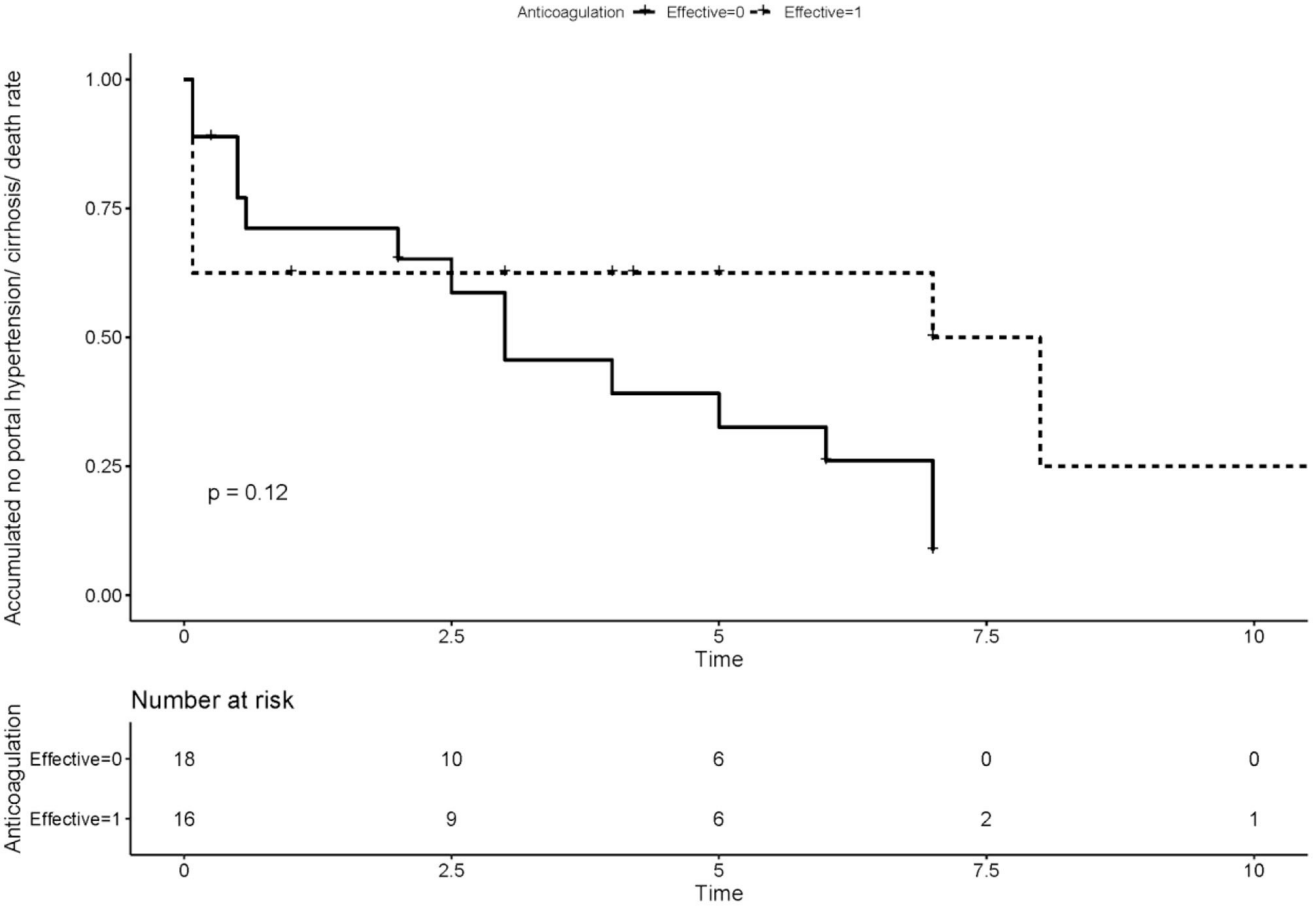
Difference of accumulated no comprehensive adverse events (portal hypertension, cirrhosis, and death) rate between groups receiving effective anticoagulation (*n* = 16) and not (*n* = 18). Effective anticoagulation was defined as immediate and sufficient anticoagulant therapy for at least 6 months with a target international normalized ratio (INR) of 2.0–3.0 from the time of portal vein thrombosis (PVT) diagnosis.

We compared the rate of aPLs, complications and treatment between groups of recanalization and no recanalization in APS patients with PVT (Table 4). There is a significant difference in the rate of effective anticoagulation between groups (*p* < 0.005), which means effective anticoagulation could increase the rate of recanalization. Survival analysis revealed that effective anticoagulation could increase recanalization rate (Figure 1) significantly (log-rank *p* = 0.001), with a lower mortality rate (log-rank *p* = 0.045; Figure 2). There was no significant difference in accumulated no portal hypertension/ cirrhosis rate (log-rank *p* = 0.32; Figure 3) and comprehensive adverse events (portal hypertension, cirrhosis, and death) rate (log-rank *p* = 0.12; Figure 4) between the effective anticoagulation group and invalid anticoagulation group.

**Table 4:** The rate of aPLs, complications and treatment between groups of re-canalization and no re-canalization in APS patients with PVT.

	**Complete/Partial re-canalization (*****n*** **= 17)**		**Recurrence/No re-canalization/Death (*****n*** **= 17)**		***P***
	***n***	**%**	***n***	**%**	
Acute	4	23.53	7	41.18	0.465
Gastrointestinal bleeding	2	11.76	3	17.65	1
Portal hypertension	9	52.94	8	47.06	0.732
Liver cirrhosis	4	23.53	5	29.41	1
Effective anticoagulation	14	82.35	2	11.76	** <0.001**
ACL	10	58.82	10	58.82	1
aβ2GP1	11	64.71	13	76.47	0.708
LAC	9	52.94	10	58.82	0.73
Splenomegaly	12	70.59	10	58.82	0.721
Blood system involvement	10	58.82	12	70.59	0.721

*Values in bold are statistically significant at p < 0.05*.

*LAC, lupus anticoagulant; ACL, anticardiolipin; antiβ2GPI, anti-β2-glycoprotein I*.

## Discussion

This is the first case-control study of the clinical characteristics and prognosis of PVT in APS patients. We found that PVT could be the first thrombotic event of APS, usually had insidious onset with atypical clinical symptoms and easily be misdiagnosed. Clinicians should pay more attention to APS patients combined with PVT. For patients with APS, the double aPLs positive, thrombocytopenia, and inflammation could be the associated factors of PVT. Early diagnosis and anticoagulation treatment can bring thrombus re-canalization thereby significantly improving the prognosis, with a lower mortality rate.

PVT could be the first thrombotic event of APS and PVT is not always symptomatic or result in complications like variceal bleeding and ascites (13). In our study, 21 patients had PVT as the first thrombotic event of APS. And seven patients were asymptomatic. Most of them were underdiagnosed or misdiagnosed at first. In other words, in cases recognized at a stage of cavernoma, the initial episode of portal venous thrombosis probably escaped attention, because signs and symptoms were mild or non-specific or because of inadequate imaging studies. Harmanci et al. (13) reported that the most common presentation of PVT is variceal bleeding followed by pancytopenia due to hypersplenism. It may be a good practice to select the potential patients according to their co-morbid conditions, degree of thrombocytopenia, and the condition of varices. Subtle laboratory abnormalities could lead to missed diagnoses and symptomatic portal hypertension is often indicative of the late stage of the PVT. Fortunately, with the advent and wide distribution of USG and Doppler-USG, the condition is becoming diagnosed earlier and a patient presenting with ascites (which is a late finding in course of PVT) is almost not seen. Esophageal and gastric varices related bleeding contribute to the most important cause of morbidity and hospitalization in this group of patients. Different from cirrhotic patients, the risk of variceal bleeding is much lower (14).

We found that double aPLs positive is an associated factor of PVT in APS patients. The presence of aPLs is associated with an increased risk of arterial and venous thrombosis, thrombocytopenia, and recurrent abortions (15). Two large series found aPL in 4 and 11% of patients with PVT (2, 3). There were two reports of PVT with APS based on the presence of LAC (8, 10). However, Janseen et al. (16) found LAC in 4.7% of 42 patients with Budd Chiari syndrome but none of 92 patients with PVT. Austin et al. found that IgA aCL may trigger thrombosis in small portal vein radicles, which drain the inflamed small intestine, leading to liver injury with consequent hyperplasia of the surrounding tissue (17). A meta-analysis (18) found that the risk of Budd–Chiari syndrome and non-cirrhotic PVT might be increased by positive IgG aCL but not IgM aCL, LAC, aβ2GPI, or β2GPI ox-LDL. But the association between aPLs and PVT in liver cirrhosis was unclear (19). The pathogenesis of aPL induced thrombosis is complex and involves the activation of platelet and neutrophil and injury of the endothelium, finally resulting in an abnormal coagulation cascade (15). 44% of “triple-positive” APS patients will develop recurrent thrombosis over a 10-year follow-up period, even with the majority being prescribed anticoagulants (20).

Our study also shows that thrombocytopenia and inflammation (hsCRP) were associated factors of PVT in APS patients. The direct binding of aPLs could lead to platelet activation and aggregation, which eventually leads to thrombocytopenia (21). The thrombosis process also consumes a large number of platelets. Cirrhosis, hypersplenism, bone marrow hematopoietic inhibition, and drugs (such as heparin) may also be involved in the process of thrombocytopenia. Therefore, thrombocytopenia has no protective effect on thrombosis. It means that hemorrhage and hypercoagulability exist at the same time, which is a high-risk factor for recurrence of thrombus and requires more attention in clinical practice. In our study, elevated CRP is an independently associated factor of PVT. A large number of studies have shown a close relationship between inflammatory status and venous thrombosis. We have already found that lupus patients with higher hsCRP have a high risk of pulmonary embolism (22). The inflammatory status could lead to pro-coagulant disorders, affect vascular homeostasis, or elevate blood coagulability through decreased blood flow speed (23), all of which contributed to the thrombotic events.

The outcome of PVT might vary from one patient to another and depend on early anticoagulation and sustained re-canalization of the portal and mesenteric veins. In the current study, 14 in 16 patients receiving effective anticoagulation got complete or partial re-canalization. Re-canalization of the portal vein on anticoagulant therapy could also prevent the development of portal hypertension (13). There have been reports of re-canalization of PVT after anticoagulation (7). However, the clinical setting of hypersplenism with low platelet counts combined with esophageal varices raises concerns about the safety of anticoagulation. A retrospective analysis (14) got the conclusion that anticoagulation was not found to be a risk factor for bleeding in non-cirrhotic PVT, while no anticoagulation resulted in more thrombotic recurrences as expected. Pharmacological prophylaxis of early anticoagulation can also decrease the incidence of PVT in cirrhotic patients (24). Anticoagulation is also recommended for APS patients with thrombotic manifestations to prevent recurrence or extension of thrombosis (25). According to current recommendations, APS patients with venous thromboembolism are best treated with standard-intensity oral anticoagulation at a target INR of 2.0–3.0 (26). However, since PVT patients, especially those with liver cirrhosis, also have a high risk of bleeding, the most suitable INR still needs more clinical research to finalize. The duration of anticoagulation has been a topic of debate; some authors recommend anticoagulation for 3–6 months in patients with a first venous thromboembolic event and a transient/reversible precipitating factor and in whom aPL becomes negative over time (27). However, some studies have shown that the risk of relapse events is significantly increased after stopping anticoagulation therapy in those with aPL (28). The choice of anticoagulant type is also a problem. The possibility of direct oral anticoagulants (DOACs) as secondary thromboprophylaxis in APS patients has been controversial. The results of open-label RCT (TRAPS trial) failed to demonstrate non-inferiority of the DOAC rivaroxaban to dose-adjusted vitamin K antagonists (VKAs) for thrombotic APS, as well as showing an increased risk of stroke with rivaroxaban (29). The results of the TRAPS study led EULAR to recommend against the use of rivaroxaban in APS patients with triple aPL positivity and a history of arterial thrombosis (30).

Our research had several limitations. The major limitation of the study is the retrospective design. Some irregular anticoagulation therapy cannot be fully reported. We only tested the total titer of the antiphospholipid antibodies, but not the specific IgG, IgA, and IgM type. Secondly, we study a small number of patients due to the low incidence of APS-PVT. Despite these limitations, our study is the first study reporting the outcome of anticoagulation therapy in APS-PVT patients. We still need larger prospective studies in the future to ensure the association between re-canalization and anticoagulation to support the recommendation of early anticoagulation therapy.

In conclusion, portal vein thrombosis is a rare and severe subtype of APS with a poor prognosis. The disease usually had insidious onset with atypical clinical symptoms and easily be misdiagnosed. Those APS patients with abdominal symptoms should be paid more attention. We should pay more attention to patients with PVT and we suggest patients with PVT screen aPLs routinely. Early diagnosis and anticoagulation treatment can bring thrombus re-canalization thereby significantly improving the prognosis.

## Data Availability Statement

The original contributions presented in the study are included in the article/supplementary material, further inquiries can be directed to the corresponding author/s.

## Ethics Statement

The studies involving human participants were reviewed and approved by the Ethics Committee of Peking Union Medical College Hospital. The patients/participants provided their written informed consent to participate in this study.

## Author Contributions

HY, JZ, ML, and XZ designed the study. HY, JZ, CH, XT, ML, and XZ collected the data and performed research. HY and JZ carried out data analyses. HY drafted the manuscript. JZ and ML helped to explain the critical points in the manuscript. All authors approved the final version of the manuscript.

## Conflict of Interest

The authors declare that the research was conducted in the absence of any commercial or financial relationships that could be construed as a potential conflict of interest.
